# Factors for healthcare utilization and effect of mutual health insurance on healthcare utilization in rural communities of South Achefer Woreda, North West, Ethiopia

**DOI:** 10.1186/s13561-018-0200-z

**Published:** 2018-08-22

**Authors:** Hiwot Tilahun, Desta Debalkie Atnafu, Geta Asrade, Amare Minyihun, Yihun Mulugeta Alemu

**Affiliations:** 1 School of Public Health, College of Medicine and Health Sciences, Bahir Dar University, Bahir Dar City, Ethiopia; 2 Curative and Rehabilitative Core Process, Amhara Regional Health Bureau, Bahir Dar City, Ethiopia; 3 Institute of Public Health, College of Medicine and Health Sciences, University of Gondar, Gondar City, Ethiopia

**Keywords:** Insurance, Enrolment, Effect, Mutual, Health, Achefere, Ethiopia

## Abstract

**Objective:**

To identify factors for healthcare utilization and to describe effect of Mutual Health Insurance on health service utilization in rural community in South Achefer, North West Ethiopia.

**Methods:**

Across-sectional study was conducted. A total of 652 households consented to participate in the study (326 insured and 326 uninsured households). Propensity score matching was used to explain possible differences in the baseline variables between enrolled and un-enrolled households. Logistic regression analysis was used to identify factors for healthcare utilization.

**Results:**

Healthcare utilization among insured households was 50.5% (95% CI: 44.8%, 56.2%). Whilst among uninsured households, healthcare utilization was 29.3% (95% CI: 24.11, 34.47). In general, the overall healthcare utilization was 39.89% (95% CI: 35.7, 43.8). The overall increase in patient-attendance given illness among insured households was 25.2% higher compared with uninsured (*t* = 4.94, 95% CI: 0.145, 0.359). Educated (primary and above) (AOR = 1.84; 95% CI: 1.14, 2.98), chronic patient (AOR = 1.86; 95% CI: 1.13, 3.06), first choice was health facilities at the point of illness (AOR = 6.33; 95% CI: 2.97–13.51), rich (AOR = 2.1; 95%CI: 1.29, 3.43), and insured (AOR = 2.16; 95% CI: 1.45, 3.23) were independently associated with increased healthcare utilization.

**Conclusion:**

Enrolment to mutual health insurance increases healthcare utilization. Presence of illness in the households, household earnings, educational status, first choice of treatment at point of illness, and membership to Mutual Health Insurance scheme should be targeted during escalating of healthcare utilization.

## Background

Globally an approximate of 44 million households (150 million people) faced catastrophic healthcare expenditure [1]. Each year, a total of 25 million households (100 million people) are pushed into deep poverty because of out of pocket healthcare spending [2]. Over 90% of healthcare expenditure catastrophes occurred in resource limited settings [3, 4].

Ethiopia is one of the highest disease burden countries [5] where utilization of facility based healthcare is very low [6]. Low level healthcare utilization might be linked with impoverished healthservices [7, 8]. Healthcare utilizations are also overwhelmed by inadequate and low quality healthcare because of limited resources and absence of dedicated healthcare financing scheme [9].

Out of pocket healthcare expenditure is one of the major problems in resource limited countries like Ethiopia [10, 11]. About 37% of the national healthcare expenditure in Ethiopia was covered due to out of pocket payments [11]. As a result of this direct way of expense, most patients were unable to afford the amount of financial expenditures for utilizing a given healthcare. Hence, they were enforced to take self-administered local medicines, and go to traditional healers or simply waiting the last date of their alive [12]. However, prepayment health insurance scheme; a best solution for financial barriers of payments made at point of healthcare use; addressed healthcare financial challenges [13]. There for, moving away from out of pocket charges for healthcare is an important step towards averting the financial ruin associated with medical care [14, 15].

Furthermore, for the last several years, unimproved healthcare services and financial burdens of healthcare are the main issues of Ethiopian people which associated with low coverage of health insurance, below 1% [2, 11]. According to Ethiopian demographic and health survey (EDHS) 2016, more than 95% of females and 94% of males were not also enclosed by any type of health insurance. Only about 1.2% of the citizens can access health insurance from both private and public agencies [13]. It is because of the fact that the fifth national health accounts of the country recommended a need to mobilize more resources for health to improve the quality, equity and access to healthcare. This has been made the focus of the national health sector transformation plan of 2015/16–2019/20. Consequently, the government of Ethiopia is currently undertaking strategies to increase government budget in the sector [11] via healthcare financing [2].

After the strategies, the following financial protection schemes were introduced: systematizing fee waivers system; revenue retention and utilizations; standardizing exemption services and initiations of health insurance [mutual health insurance (MHIS) and social health insurance (SHI)]. However, social health insurance which would suppose to cover 10.46% of the population in the formal sectors is a compulsory one. Whilst, mutual health insurance scheme was proposed to address 83.6% of Ethiopian citizens who are engaged in informal sectors; mainly the rural residents [11]. The policy agenda of health insurances coverage is aimed to deliver equitable access; to sustainable quality of healthcare, to decrease financial barrier; and to develop social inclusion for households in the health sector. Unlike social health insurance, membership in mutual health insurance scheme is based on voluntary decisions which are made by households. A household contributed an amount of 10.4 US$ per annum to join mutual health insurance scheme. Nonetheless, the amount of money contributed to the mutual health insurance might vary from year to year or based on nature of household’s income. The mutual health insurance scheme benefit package covers all outpatient and inpatient services at all levels of the health facilities except for dentures, eyeglasses, and cosmetic healthcare services**.** In Ethiopia, despite remarkable benefits, less than 1% of people are enclosed by social health insurance, and the same percentage covered by employer-based insurance [9, 12].

The effects of health insurance on healthcare utilization and factors for healthcare utilization are not well described in resource limited settings particularly Ethiopia. This study aims to determine effects of mutual health insurance on healthcare utilization and to identify factors for healthcare utilization in rural community in South Achefer, North West Ethiopia.

## Methods

### Study design and setting

We conducted a cross-sectional study from February to March, 2016 in rural community of South Achefer, Northwest Ethiopia. In 20 kebeles, a total of 36,204 households were identified; of which 12,612(35%) households were enrolled to mutual health insurance [16].

### Goals and challenges of the empirical analysis

House to house, community-based survey was conducted in South Achefer district. The objectives of this study were to determine healthcare utilization among insured and uninsured households and to identify factors linked with healthcare utilization. The main methodological challenges could be selection bias and variable omission because of unobserved heterogeneity during matching. Heterogeneity is a variation across individual units of observations, and the variation can’t be observed. The term heterogeneity refers to differences across the units being studied. For example, in an insurance model, members with non-members are assumed to differ from one another is said to have heterogeneous agents. We might have an omitted variable (as is often with cases of causal inference and selection bias) that we say we have endogeneity caused by unobserved heterogeneity. The effect of unobserved heterogeneity is endogeneity that is correlation between explanatory variables and error term. Unobserved heterogeneity is one instance in where correlation between observables and unobservable may be expected. In addition to the observed variables under study, there are other relevant variables which are unobserved, but correlated with the observed variables. Mutual health insurance scheme was voluntary based which could lead people to self-select themselves in to a particular group (either being insured or uninsured) [7, 14, 15]. Therefore, we applied a technique called propensity score matching to account for potential differences in background characteristics between insured and uninsured so that difference in the outcome of interest could be attributed to the intervention itself.

### Sampling and study participants

The study was designed to have 80% statistical power in order to get adequate sample size for justifiable statistical significant given efficient resource utilization. The level of significance was taken 5%. The ratio of insured to uninsured was 1:1. Assuming the proportion of health service utilization was 35% for insured households and 20% for uninsured household [17]. The sample size was estimated to be 296 for insured and 296 for uninsured households. The non-response rate of 10% was taken. The design effect was determined to be two for having two stages in selecting study subjects, the resulting sample size was 652 (326 insured and 326 uninsured households). The sample size was calculated for different objectives and the largest sample estimate was taken.

### Sampling procedure

In this study, for large clusters of population, multi-stage sampling procedure was appropriate. Multi-stage sampling could divide large clusters of population into smaller clusters in several stages in order to make primary data collection manageable and cost wise. Of 20 Kebeles, six Kebeles were randomly selected from the South Achefer district. A total of 652 households were identified from six Kebeles. Proportional allocation of households per kebele was employed. Sampled households (insured and uninsured) were selected systematically from each Kebele.

### Data collection and study variables

Ten trained diploma graduate data collectors conducted face to face interviews with household heads. The data collection tools were structured questionnaires that were prepared in English, translated into Amharic and back translated to check its consistency. Pre-test was conducted for reliability and ease of understanding. We measured all variables at the household level. Enrollment to mutual health insurance was at the household level. Health services utilization was the dependent variable. Independent variables were age, sex, education, family size, distance from health institutions, insurance membership, wealth index, first choice place for treatment, chronic illness and health seeking behavior.

### Operational definitions

Utilization of healthcare was defined as health facilities visits by members of households at least once in the last 6 months for health services (diagnostic or treatment). Perceived health status was respondent’s report about their health status that was assigned numerical values according to the following scale: very good = 5, good = 4 medium =3 poor =2 and very poor = 1. Perceived quality of care was the extent of respondent’s view on the quality of healthcare delivery; it was measured by Likert scale questions. Chronic illness is a disease condition that lasts more than three months. Household wealth index was measured by using principal component analysis and categorized as poor, medium and rich. Households indexed in the mustered book of mutual health insurances schemes were recruited as insured while households which were not indexed to mutual health insurance schemes were recruited as uninsured.

### Data analysis

Data were entered into Epi-info and analysis was performed by using STATA. Frequencies and proportions were used to describe the respondents in relation to the studied variables. To identify factors associated with healthcare utilization, logistic regression models were used. A bi-variable logistic regression model was fitted for each explanatory variable to identify the existence of associations among candidate and outcome variables. Multivariable logistic regression analysis (backward stepwise) was used to identify independent predictors for healthcare utilizations. All variables whose crude odds ratios greater than 0.2 in the bi-variable logistic regression model were fitted in to multivariable logistic regression model. An odds ratio with 95% confidence interval was used to measure the strength of association and identify statistically significant results. Propensity score matching was used to explain possible differences in the baseline variables between enrolled and un-enrolled households. In this data set a good-quality matching was observed since mean absolute bias was 4% which was < 5%. Therefore, the variation in healthcare utilization was due to average impact of mutual health insurance itself.

### Ethical considerations

Ethical approval from the University of Gondar Ethics committee was obtained. In addition, ethical endorsement from Amhara Regional Health Bureau Research Core Process Unit was approved. Written permission to conduct the study was obtained from each Kebele administration involved in the study. The data collectors took written informed consent from household heads.

## Results

### Socio-demographic and other characteristics of respondents

A total of 594 households participated in the study with a response rate of 91.1%. The average age of the respondents was 47.5 years (±14.6SD). Table 1 presents socio-demographic characteristics of study participants, enabling and needs factors. Among study subjects, more female household heads (47%) were utilizing healthcare services than male household heads (36%). Among respondents who were not able to write and read, higher percentage (68%) were not utilizing health care service than respondents who were able to write and read (51%). A considerable percentage of household heads who traveled less than five kilometers to reach the nearest health institutions (43%) were utilizing health services than household heads who traveled five kilometers and above (37%). Higher proportion of respondents with chronic illness in the households (52%) were utilized the health services than households without chronically ill respondents (37.4%) (Table 1).

**Table 1:** Presentation of characteristics of study participants by health service utilization in rural communities of South Achefer, March 2016 (*n* = 594)

Variables	Health service utilization	
	No (%)	Yes (%)
Sex of respondents		
Male	249(64)	143(36)
Female	108(53)	94(47)
Educational level		
Unable to read & write	259(68)	120(32)
Able to read & write	42(51)	41(49)
Primary education and above	56(42)	76(58)
Family size		
< 5	159(63)	93(37)
≥ 5	198(58)	144(42)
Distance from health facilities in kilo meters		
< 5	184(57)	137(43)
≥ 5	173(63)	100(37)
Chronic illness		
No	307 (62.6)	183 (37.4)
Yes	50 (48)	54(52)
Household wealth index		
Poor	140(70.7)	58(29.3)
Medium	120(60.6)	78(39.4)
Rich	97(48.9)	101(54.1)
First choice place for treatment at the point of illness		
Health institution	273(54.6)	227(55.4)
Traditional healer	84(89)	10(11)
Perceived health status		
Poor	64(71)	26 (29)
Medium	185 (67)	92 (33)
Good	81 (58)	58(42)
Very good	27(31)	61(69)
Enrollment to mutual health insurance		
No	210(72)	87(28)
Yes	147(49)	150(52)

### Multilevel logistic regression

Respondents, whose educational level was primary and above, were more likely to utilize health service (AOR = 1.84; 95% CI: 1.14, 2.98) than respondents who did not write and read. Households with presence of a chronic patient were more likely to utilize health services (AOR = 1.86; 95% CI: 1.13, 3.06) than its counter parts. Households, whose wealth index was categorized as rich, were more likely to utilize health service (AOR = 2.1; 95%CI: 1.29, 3.43) than households, whose wealth index was categorized as poor. Respondents whose first choice was health institution for treatment at the point of illness were more likely to utilize health service (AOR = 6.33; 95% CI: 2.97, 13.51) than household heads whose first choice was traditional healer. Respondents with poor perceived health status were less likely to utilize health service (AOR = 0.33; 95% CI: 0.18, 0.61) than respondents with perceived health status of very good. Households who enrolled to mutual health insurance scheme were more likely to utilize health service (AOR = 2.16; 95% CI: 1.45, 3.23) compared to households who did not enrolled to mutual health insurance scheme (Table 2).

**Table 2:** Factors associated with utilization of health service in South Achefere, March 2016 (*N* = 594)

Variables	Health service utilization		COR (95% CI)	AOR (95% CI)
	No	Yes		
Sex of respondents				
Male	249	143	1.00	1.00
Female	108	94	1.52 (1.07–2.14)	1.47 (0.97–2.22)
Educational level				
Unable to read & write	259	120	1.00	1.00
Able to read &write	42	41	2.11 (1.30–3.41)	1.75 (1.003–3.06)^a^
Primary education and above	56	76	2.93 (1.95–4.40)	1.84 (1.14–2.98)^a^
Family size				
< 5	159	93	1.00	1.00
≥ 5	198	144	1.35(0.92–1.98)	1.31(0.83–2.07)
Distance from health facilities in kilo meters				
< 5	184	137	1.29(0.93–1.79)	1.47(0.98,2.2)
≥ 5	173	100	1.00	1.00
Chronic illness				
No	307	183	1.00	1.00
Yes	50	54	1.81 (1.18–2.77)	1.86 (1.13–3.06)^a^
Household wealth index				
Poor	140	58	1.00	1.00
Medium	120	78	1.57 (1.03–2.384)	1.52(0.94–2.46)
Rich	97	101	2.52(1.66–3.8)	2.1(1.29–3.43)^a^
First choice place for treatment at the point of illness				
Health institution	273	227	1.00	6.33(2.97–13.51)^a^
Traditional healer	84	10	6.99(3.54–13.77)	1.00
Perceived health status				
Poor	64	26	0.15 (0.07–0.29)	0.18 (0.09–0.4)^a^
Medium	185	92	0.22 (0.13–0.37)	0.28 (0.16–0.51)^a^
Good	81	58	0.32 (0.18–0.56)	0.33 (0.18–0.61)^a^
Very good	27	61	1.00	1.00
Enrolment to mutual health insurance				
No	210	87	1.00	1.00
Yes	147	150	2.46(1.76–3.45)	2.16 (1.45–3.23)^a^

*COR* Crude odds ratio

*AOR* Adjusted odds ratio

^a^= Independently significant at α 0.05

### Effect of mutual health insurance on health service utilization

Before matching was computed, healthcare utilization is significantly higher in households which were enrolled to mutual health insurance (50.5%) than households which were not enrolled to mutual health insurance (29.3%) (*t* = 5.4, *p*-value< 0.001). To minimize self-selection bias of insurance uptake, nearest neighborhood matching was done between who were enrolled (treated) to mutual health insurance (*n* = 297) and who were not enrolled (control) to mutual health insurance (*n* = 218). The average treatment effect on the treated group was 0.252 (*t* = 4.94, 95% CI: 0.14–0.35). Being a member to mutual health insurance contributed approximately 25.2% point increase healthcare utilization. The average incremental effect on healthcare utilization was because of enrollment to mutual health insurance scheme (Table 3).

**Table 3:** The average treatment effect of mutual health insurance on healthcare utilization, South Achefer Woreda, March 2016 (*n* = 594)

Outcome	Means before matching		Difference	*p*-value	ATT	S. E	T	95% CI		No. of cases	
	Insured	Uninsured								Insured	Uninsured
HSU	0.505	0.293	0.212	< 0.001	0.252	0.05	4.9	0.14	0.35	297	218

*HSU* Health service utilization, *ATT* Average treatment effect on treated, *S.E* Standard error, *t* Student’s t distribution

## Discussion

Enrolment to healthcare insurance increases use of healthcare in various settings [15, 18]. In Ethiopia (37%) of households are reliance on out of packet spending; health insurance coverage remains low [11, 13, 15]. This study determined the effect of mutual health insurance on healthcare utilization and identified factors associated with healthcare utilization.

Health insurance scheme is a crucial strategy for financial protection of many households [14]. Mutual health insurance scheme prevents financial hardship during illness. Mutual health insurance scheme increases healthcare utilization [13]. This study shown that the overall health service utilization rate in the past 6 month was 39.9% (95% CI: 35.7,43.8) which was consistent with the study conducted in Amhara region (38.7%) [19]. However, our study finding was lower than the study finding of Federal Ministry of Health (48%) and the study finding in Jimma Zone (54.76%) [20]. Difference in effect of mutual health insurance on healthcare utilization could be attributed to difference in health seeking behaviour, severity of illness, healthcare coverage and perception to healthcare quality of various settings.

Before matching, the percentage value of healthcare utilization is significantly higher in mutual health insurance enrolled group (50.5%) than the non-enrolled (29.3%) (*t* = 5.4, *p*-value< 0.001). This finding was consistent to study findings in Burkina Faso; the percentage of healthcare utilization was 37% among insured and 12% among uninsured. Similarly, the study showed that the proportion of unmatched women who visited at least one antenatal care was higher among insured women than uninsured women [15]. The matching estimator of this study showed that household participated in the mutual health insurance contributed a 25.2% (*t* = 4.94; 95% CI: 0.14, 0.35) increase in healthcare utilization. This finding was higher in magnitude than the finding in Rwanda (15%) [21].

Community health insurance program in Ethiopia aimed to enhance healthcare utilization. In this study mutual health insurance increased healthcare utilization. This was consistent with the study finding in Burkina Faso, Nuna district where the level of health service utilization were higher among insured groups [22]. Community health insurance motivates participants to attend more healthcares.

Low income earned households were less likely to utilize healthcare than households earned higher income [23–25]. In this study, households which were categorized as rich were more likely utilize healthcare than households whose wealth index was categorized as poor. Our study finding was consistent with a study conducted in Rwanda [26] and China [27] which showed that high income farmers were more likely to utilize outpatient services than low income farmers. This is because of the perceived cost of premium and treatment. Respondents whose wealth index rich were able and afford to pay the premium for enrolment as reduction of marginal utility of wealth and likely to consume more healthcares due to moral hazard. In addition, higher income groups, who feared loss of their money due to illness (indirect cost of illness or production loss) had shown alert for increased demand in healthcare utilization by contributing premium in their insurance [28].

In this study, the utilization of health services by respondents observed their health poor was too low (28.9%). This is lower than reported in Amhara region (38.7%) [19], in Jimma (52.9%) [20]. The probable reason for not utilizing modern healthcare service by those perceived their health poor was a need for treatment made at home due to traditional healers followed by reduction in cost of transportation and treatment given shortage of money. There were a massive known practices of traditional medicines found at home made off local knowledge from herbal leaf given by traditional healers [29]. It is also possible to note that the community healthcare seeking behavior is compromised and result in lower adverse selection given an efficient and effective insurance program.

A study finding in Ghana identified that enrollment to national health insurance increase their preference of healthcare provided at healthcare facility than at home or traditional healers [30]. The same is also true in the case of study conducted in Burikina Faso [31]. Similarly, our research report showed that respondents who were enrolled to mutual health insurance were less likely to visit traditional healer compared with uninsured. Enrollments into mutual health insurance could ensure healthcare preference to facility based healthcare than traditional healers [32]. Uninsured households refrain from facilities based healthcare (hospital, health center) and might increase attendance for traditional healer given lower service cost of healings and transportation compared to healthcare facilities did [32]. In general, the decision to visit a traditional healer is not an easily job of whither to select a given provider or not, nonetheless it is the matter of choice made between able and afford to buy a given healing.

### Limitations

As we conducted cross-sectional study, factors do not establish temporal relationship. Selection bias due to matching may result in variable omission; unobserved heterogeneity could be exhibited. An important source of potential bias did exist as participation. In general, mutual health insurance scheme was voluntary which could lead people to self-select themselves in to a particular group (either being insured or uninsured). This results in endogeneity.

## Conclusion

Enrolment to mutual health insurance increases healthcare utilization. Presence of illness in the households, household earnings, educational status, first choice of treatment at point of illness, and membership to mutual health insurance scheme should be targeted during escalating of healthcare utilization.

## Abbreviations

AOR: Adjusted odds ratio
CI: Confidence interval
COR: Crude odds ratio
EDHS: Ethiopian demographic and health survey
MHIS: Mutual health insurance scheme
SD: Standard deviations
SHI: Social health insurance
t: Students’ t distribution
US: United states
